# 3-(4-Chloro­phenyl­sulfon­yl)-5-isopropyl-2-methyl-1-benzofuran

**DOI:** 10.1107/S1600536811014784

**Published:** 2011-04-29

**Authors:** Hong Dae Choi, Pil Ja Seo, Uk Lee

**Affiliations:** a Department of Chemistry, Dongeui University, San 24 Kaya-dong Busanjin-gu, Busan 614-714, Republic of Korea; b Department of Chemistry, Pukyong National University, 599-1 Daeyeon 3-dong, Nam-gu, Busan 608-737, Republic of Korea

## Abstract

In the title mol­ecule, C_18_H_17_ClO_3_S, the 4-chloro­phenyl ring makes a dihedral angle of 77.03 (5)° with the mean plane of the benzofuran fragment. In the crystal structure, the mol­ecules are linked by weak inter­molecular C—H⋯O hydrogen bonds and C—H⋯π inter­actions

## Related literature

For the pharmacological activity of benzofuran compounds, see: Aslam *et al.* (2009 ▶); Galal *et al.* (2009 ▶); Khan *et al.* (2005 ▶). For natural products with benzofuran rings, see: Akgul & Anil (2003 ▶); Soekamto *et al.* (2003 ▶). For the structures of related 3-aryl­sulfonyl-5-isopropyl-2-methyl-1-benzofuran derivatives, see: Choi *et al.* (2008 ▶, 2010 ▶).
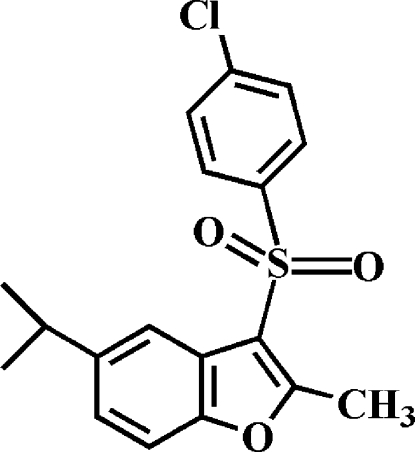


## Experimental

### 

#### Crystal data

C_18_H_17_ClO_3_S
*M* *_r_* = 348.83
Monoclinic, 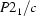

*a* = 11.4851 (4) Å
*b* = 11.3194 (4) Å
*c* = 13.0600 (4) Å
β = 101.475 (2)°
*V* = 1663.92 (10) Å^3^
*Z* = 4
Mo *K*α radiation
μ = 0.37 mm^−1^
*T* = 296 K
0.29 × 0.20 × 0.18 mm

#### Data collection

Bruker SMART APEXII CCD diffractometer
Absorption correction: multi-scan (*SADABS*; Bruker, 2009 ▶) *T*_min_ = 0.660, *T*_max_ = 0.746
14572 measured reflections
3643 independent reflections
3109 reflections with *I* > 2σ(*I*)
*R*_int_ = 0.040

#### Refinement

*R*[*F* ^2^ > 2σ(*F* ^2^)] = 0.043
*wR*(*F* ^2^) = 0.120
*S* = 1.08
3643 reflections
211 parameters
1 restraint
H-atom parameters constrained
Δρ_max_ = 0.67 e Å^−3^
Δρ_min_ = −0.65 e Å^−3^

### 

Data collection: *APEX2* (Bruker, 2009 ▶); cell refinement: *SAINT* (Bruker, 2009 ▶); data reduction: *SAINT*; program(s) used to solve structure: *SHELXS97* (Sheldrick, 2008 ▶); program(s) used to refine structure: *SHELXL97* (Sheldrick, 2008 ▶); molecular graphics: *ORTEP-3* (Farrugia, 1997 ▶) and *DIAMOND* (Brandenburg, 1998 ▶); software used to prepare material for publication: *SHELXL97*.

## Supplementary Material

Crystal structure: contains datablocks global, I. DOI: 10.1107/S1600536811014784/su2271sup1.cif

Structure factors: contains datablocks I. DOI: 10.1107/S1600536811014784/su2271Isup2.hkl

Additional supplementary materials:  crystallographic information; 3D view; checkCIF report

## Figures and Tables

**Table 1:** Hydrogen-bond geometry (Å, °) *Cg*1 is the centroid of the C2–C7 benzene ring.

*D*—H⋯*A*	*D*—H	H⋯*A*	*D*⋯*A*	*D*—H⋯*A*
C12—H12*C*⋯O3^i^	0.96	2.57	3.489 (3)	160
C11—H11*B*⋯*Cg*^ii^	0.96	2.81	3.562 (3)	136

Symmetry codes: (i) 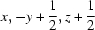; (ii) 
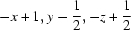
.

**Fig. 1.**
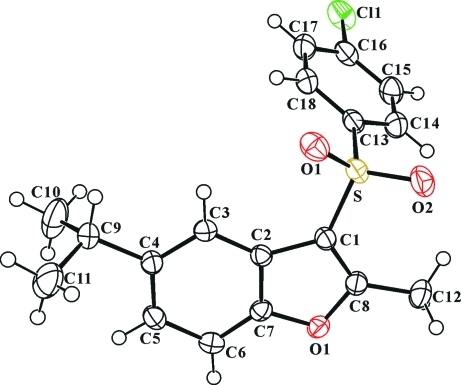
The molecular structure of the title compound with the atom numbering scheme. Displacement ellipsoids are drawn at the 50% probability level. H atoms are presented as a small spheres of arbitrary radius.

**Fig. 2.**
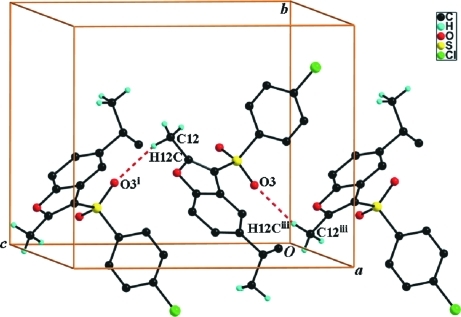
A view along the a axis of the C—H···O interactions (dotted lines) in the crystal structure of the title compound. [See Table 1 for details; Symmetry codes: (i) x, -y + 1/2, z + 1/2; (iii) x, -y + 1/2, z - 1/2]

**Fig. 3.**
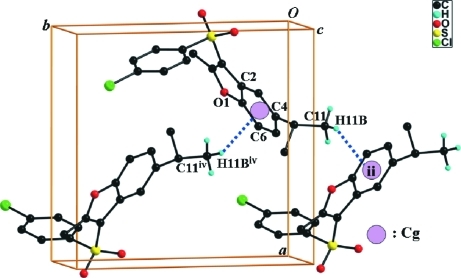
A view along the c axis of the C—H···π interactions (dotted lines) in the crystal structure of the title compound. [See Table 1 for details; Symmetry codes: (ii) - x + 1, y -1/2, - z + 1/2; (iv) -x + 1, y + 1/2, -z + 1/2]

## supplementary crystallographic information

### Comment

Many compounds containing a benzofuran ring have recently drawn considerable attention due to their interesting pharmacological properties such as antibacterial and antifungal, antitumor and antiviral, and antimicrobial activities (Aslam *et al.*, 2009; Galal *et al.*, 2009; Khan *et al.*, 2005). These compounds occur in a wide range of natural products (Akgul & Anil, 2003; Soekamto *et al.*, 2003). As a part of our ongoing program of the substituent effect on the solid state structures of 3-arylsulfonyl-5-isopropyl-2-methyl-1-benzofuran analogues (Choi *et al.*, 2008, 2010), we report herein the crystal structure of the title compound.

In the title molecule (Fig. 1), the benzofuran unit is essentially planar, with a mean deviation of 0.007 (1) Å from the least-squares plane defined by the nine constituent atoms. The dihedral angle formed by the 4-chlorophenyl ring and the mean plane of the benzofuran fragment is 77.03 (5)°. The crystal packing (Fig. 2) is stabilized by weak intermolecular C—H···O hydrogen bonds between a methyl H atom and the O atom of the sulfonyl group (Table 1; C12—H12C···O3^i^). The crystal packing (Fig. 3) is further stabilized by intermolecular C—H···π interactions between a methyl H atom of the isopropyl group and the benzene ring of an adjacent molecule (Table 1; C10—H11B···Cg^ii^, Cg is the centroid of the C2–C7 benzene ring).

### Experimental

77% 3-chloroperoxybenzoic acid (560 mg, 2.5 mmol) was added in small portions to a stirred solution of 3-(4-chlorophenylsulfanyl)-5-isopropyl-2-methyl-1-benzofuran (380 mg, 1.2 mmol) in dichloromethane (40 ml) at 273 K. After being stirred at room temperature for 6 h, the mixture was washed with saturated sodium bicarbonate solution and the organic layer was separated, dried over magnesium sulfate, filtered and concentrated at reduced pressure. The residue was purified by column chromatography (benzene) to afford the title compound as a colourless solid [yield 71%, m.p. 409–410 K; *R*_f_ = 0.65 (benzene)]. Single crystals suitable for X-ray diffraction were prepared by slow evaporation of a solution of the title compound in acetone at room temperature.

### Refinement

All H atoms were positioned geometrically and refined using a riding model: C—H = 0.93 Å for aryl, 0.98 Å for methine, and 0.96 Å for methyl H atoms, with *U*_iso_(H) = 1.2*U*_eq_(C) for aryl and methine, and 1.5*U*_eq_(C) for methyl H atoms.

### Crystal data

**Table:**

C_18_H_17_ClO_3_S	*F*(000) = 728
*M**_r_* = 348.83	*D*_x_ = 1.392 Mg m^−^^3^
Monoclinic, *P*2_1_/*c*	Mo *K*α radiation, λ = 0.71073 Å
Hall symbol: -P 2ybc	Cell parameters from 5946 reflections
*a* = 11.4851 (4) Å	θ = 2.4–28.3°
*b* = 11.3194 (4) Å	µ = 0.37 mm^−^^1^
*c* = 13.0600 (4) Å	*T* = 296 K
β = 101.475 (2)°	Block, colourless
*V* = 1663.92 (10) Å^3^	0.29 × 0.20 × 0.18 mm
*Z* = 4	

### Data collection

**Table:**

Bruker SMART APEXII CCD diffractometer	3643 independent reflections
Radiation source: rotating anode	3109 reflections with *I* > 2σ(*I*)
graphite multilayer	*R*_int_ = 0.040
Detector resolution: 10.0 pixels mm^-1^	θ_max_ = 27.0°, θ_min_ = 1.8°
φ and ω scans	*h* = −14→14
Absorption correction: multi-scan (*SADABS*; Bruker, 2009)	*k* = −14→14
*T*_min_ = 0.660, *T*_max_ = 0.746	*l* = −16→16
14572 measured reflections	

### Refinement

**Table:**

Refinement on *F*^2^	Primary atom site location: structure-invariant direct methods
Least-squares matrix: full	Secondary atom site location: difference Fourier map
*R*[*F*^2^ > 2σ(*F*^2^)] = 0.043	Hydrogen site location: difference Fourier map
*wR*(*F*^2^) = 0.120	H-atom parameters constrained
*S* = 1.08	*w* = 1/[σ^2^(*F*_o_^2^) + (0.0579*P*)^2^ + 0.8346*P*] where *P* = (*F*_o_^2^ + 2*F*_c_^2^)/3
3643 reflections	(Δ/σ)_max_ < 0.001
211 parameters	Δρ_max_ = 0.67 e Å^−^^3^
1 restraint	Δρ_min_ = −0.65 e Å^−^^3^

### Special details

**Table:**

Geometry. All esds (except the esd in the dihedral angle between two l.s. planes) are estimated using the full covariance matrix. The cell esds are taken into account individually in the estimation of esds in distances, angles and torsion angles; correlations between esds in cell parameters are only used when they are defined by crystal symmetry. An approximate (isotropic) treatment of cell esds is used for estimating esds involving l.s. planes.
Refinement. Refinement of F^2^ against ALL reflections. The weighted R-factor wR and goodness of fit S are based on F^2^, conventional R-factors R are based on F, with F set to zero for negative F^2^. The threshold expression of F^2^ > 2sigma(F^2^) is used only for calculating R-factors(gt) etc. and is not relevant to the choice of reflections for refinement. R-factors based on F^2^ are statistically about twice as large as those based on F, and R- factors based on ALL data will be even larger.

### Fractional atomic coordinates and isotropic or equivalent isotropic displacement parameters (Å^2^)

**Table:**

	*x*	*y*	*z*	*U*_iso_*/*U*_eq_	
Cl1	0.26041 (5)	0.75171 (5)	−0.02985 (5)	0.04730 (19)	
S1	0.05170 (4)	0.34612 (5)	0.20282 (4)	0.02918 (16)	
O1	0.28084 (13)	0.32071 (12)	0.46858 (10)	0.0284 (3)	
O2	−0.04776 (13)	0.39127 (15)	0.24196 (13)	0.0410 (4)	
O3	0.03725 (14)	0.24367 (13)	0.13660 (12)	0.0364 (4)	
C1	0.16557 (17)	0.31448 (17)	0.30843 (15)	0.0259 (4)	
C2	0.26193 (16)	0.23122 (16)	0.30977 (14)	0.0234 (4)	
C3	0.29613 (17)	0.15214 (17)	0.23947 (15)	0.0267 (4)	
H3	0.2521	0.1454	0.1718	0.032*	
C4	0.39669 (18)	0.08375 (18)	0.27190 (15)	0.0276 (4)	
C5	0.46245 (17)	0.09257 (17)	0.37270 (14)	0.0290 (4)	
H5	0.5292	0.0452	0.3930	0.035*	
C6	0.42883 (17)	0.17475 (17)	0.44745 (14)	0.0279 (4)	
H6	0.4720	0.1827	0.5153	0.033*	
C7	0.32941 (17)	0.23906 (16)	0.41025 (14)	0.0242 (4)	
C8	0.18122 (18)	0.36468 (18)	0.40469 (16)	0.0287 (4)	
C9	0.4355 (2)	−0.0030 (2)	0.19646 (17)	0.0356 (5)	
H9	0.3747	−0.0018	0.1321	0.043*	
C10	0.5518 (3)	0.0338 (3)	0.1676 (3)	0.0635 (8)	
H10A	0.5444	0.1124	0.1395	0.095*	
H10B	0.5708	−0.0197	0.1163	0.095*	
H10C	0.6140	0.0320	0.2288	0.095*	
C11	0.4420 (3)	−0.1283 (2)	0.2381 (2)	0.0586 (8)	
H11A	0.5058	−0.1344	0.2978	0.088*	
H11B	0.4560	−0.1818	0.1848	0.088*	
H11C	0.3684	−0.1482	0.2581	0.088*	
C12	0.1135 (2)	0.4515 (2)	0.45385 (18)	0.0420 (6)	
H12A	0.0530	0.4865	0.4013	0.063*	
H12B	0.1662	0.5119	0.4874	0.063*	
H12C	0.0772	0.4124	0.5048	0.063*	
C13	0.10801 (17)	0.46178 (18)	0.13593 (16)	0.0289 (4)	
C14	0.1036 (2)	0.5775 (2)	0.17029 (17)	0.0358 (5)	
H14	0.0693	0.5944	0.2274	0.043*	
C15	0.1502 (2)	0.6673 (2)	0.11922 (19)	0.0383 (5)	
H15	0.1484	0.7450	0.1419	0.046*	
C16	0.19963 (18)	0.6399 (2)	0.03376 (18)	0.0339 (5)	
C17	0.2032 (2)	0.5257 (2)	−0.00179 (18)	0.0368 (5)	
H17	0.2363	0.5092	−0.0597	0.044*	
C18	0.15686 (19)	0.4358 (2)	0.04978 (16)	0.0336 (5)	
H18	0.1585	0.3583	0.0267	0.040*	

### Atomic displacement parameters (Å^2^)

**Table:**

	*U*^11^	*U*^22^	*U*^33^	*U*^12^	*U*^13^	*U*^23^
Cl1	0.0377 (3)	0.0432 (4)	0.0619 (4)	−0.0010 (2)	0.0123 (3)	0.0175 (3)
S1	0.0243 (3)	0.0286 (3)	0.0343 (3)	0.00487 (19)	0.0049 (2)	0.0052 (2)
O1	0.0352 (7)	0.0273 (7)	0.0242 (7)	0.0024 (6)	0.0093 (6)	−0.0011 (5)
O2	0.0282 (8)	0.0458 (10)	0.0512 (9)	0.0108 (7)	0.0136 (7)	0.0117 (8)
O3	0.0346 (8)	0.0310 (8)	0.0399 (8)	0.0000 (6)	−0.0015 (7)	−0.0003 (6)
C1	0.0260 (9)	0.0242 (9)	0.0283 (9)	0.0041 (7)	0.0076 (7)	0.0036 (8)
C2	0.0235 (9)	0.0224 (9)	0.0247 (9)	0.0009 (7)	0.0057 (7)	0.0031 (7)
C3	0.0297 (10)	0.0274 (10)	0.0222 (9)	0.0030 (8)	0.0034 (8)	−0.0001 (7)
C4	0.0303 (10)	0.0250 (10)	0.0288 (9)	0.0043 (8)	0.0092 (8)	0.0019 (8)
C5	0.0251 (9)	0.0283 (11)	0.0336 (10)	0.0041 (8)	0.0056 (8)	0.0052 (8)
C6	0.0281 (9)	0.0292 (10)	0.0246 (9)	−0.0026 (8)	0.0012 (8)	0.0031 (8)
C7	0.0282 (9)	0.0221 (9)	0.0238 (9)	−0.0024 (7)	0.0090 (7)	0.0014 (7)
C8	0.0327 (10)	0.0245 (10)	0.0319 (10)	0.0034 (8)	0.0133 (8)	0.0041 (8)
C9	0.0426 (12)	0.0338 (12)	0.0312 (10)	0.0136 (9)	0.0095 (9)	−0.0016 (9)
C10	0.073 (2)	0.0551 (18)	0.077 (2)	0.0068 (14)	0.0497 (17)	−0.0074 (15)
C11	0.096 (2)	0.0326 (13)	0.0543 (16)	0.0061 (14)	0.0326 (16)	−0.0058 (11)
C12	0.0532 (14)	0.0375 (13)	0.0400 (12)	0.0143 (11)	0.0209 (11)	−0.0020 (10)
C13	0.0253 (9)	0.0287 (10)	0.0321 (10)	0.0072 (8)	0.0041 (8)	0.0051 (8)
C14	0.0398 (11)	0.0320 (12)	0.0373 (11)	0.0078 (9)	0.0117 (9)	0.0019 (9)
C15	0.0431 (12)	0.0269 (11)	0.0455 (12)	0.0044 (9)	0.0101 (10)	−0.0010 (9)
C16	0.0254 (10)	0.0342 (12)	0.0412 (12)	0.0041 (8)	0.0045 (8)	0.0100 (9)
C17	0.0351 (11)	0.0397 (13)	0.0384 (11)	0.0087 (9)	0.0141 (9)	0.0055 (9)
C18	0.0355 (11)	0.0306 (11)	0.0354 (11)	0.0091 (9)	0.0086 (9)	0.0017 (9)

### Geometric parameters (Å, °)

**Table:**

Cl1—C16	1.735 (2)	C9—C10	1.517 (4)
S1—O2	1.4352 (15)	C9—H9	0.9800
S1—O3	1.4364 (16)	C10—H10A	0.9600
S1—C1	1.739 (2)	C10—H10B	0.9600
S1—C13	1.766 (2)	C10—H10C	0.9600
O1—C8	1.369 (2)	C11—H11A	0.9600
O1—C7	1.384 (2)	C11—H11B	0.9600
C1—C8	1.359 (3)	C11—H11C	0.9600
C1—C2	1.451 (3)	C12—H12A	0.9600
C2—C7	1.388 (3)	C12—H12B	0.9600
C2—C3	1.394 (3)	C12—H12C	0.9600
C3—C4	1.385 (3)	C13—C18	1.385 (3)
C3—H3	0.9300	C13—C14	1.388 (3)
C4—C5	1.385 (3)	C14—C15	1.381 (3)
C4—C9	1.519 (3)	C14—H14	0.9300
C5—C6	1.4551 (17)	C15—C16	1.384 (3)
C5—H5	0.9300	C15—H15	0.9300
C6—C7	1.360 (3)	C16—C17	1.377 (3)
C6—H6	0.9300	C17—C18	1.383 (3)
C8—C12	1.477 (3)	C17—H17	0.9300
C9—C11	1.516 (3)	C18—H18	0.9300
			
O2—S1—O3	119.81 (10)	C9—C10—H10A	109.5
O2—S1—C1	108.49 (9)	C9—C10—H10B	109.5
O3—S1—C1	106.86 (9)	H10A—C10—H10B	109.5
O2—S1—C13	107.93 (9)	C9—C10—H10C	109.5
O3—S1—C13	108.09 (10)	H10A—C10—H10C	109.5
C1—S1—C13	104.68 (9)	H10B—C10—H10C	109.5
C8—O1—C7	106.68 (15)	C9—C11—H11A	109.5
C8—C1—C2	107.47 (17)	C9—C11—H11B	109.5
C8—C1—S1	126.02 (15)	H11A—C11—H11B	109.5
C2—C1—S1	126.50 (15)	C9—C11—H11C	109.5
C7—C2—C3	119.10 (17)	H11A—C11—H11C	109.5
C7—C2—C1	104.54 (16)	H11B—C11—H11C	109.5
C3—C2—C1	136.35 (18)	C8—C12—H12A	109.5
C4—C3—C2	119.01 (18)	C8—C12—H12B	109.5
C4—C3—H3	120.5	H12A—C12—H12B	109.5
C2—C3—H3	120.5	C8—C12—H12C	109.5
C3—C4—C5	120.95 (17)	H12A—C12—H12C	109.5
C3—C4—C9	119.83 (18)	H12B—C12—H12C	109.5
C5—C4—C9	119.22 (17)	C18—C13—C14	120.6 (2)
C4—C5—C6	121.10 (17)	C18—C13—S1	119.47 (17)
C4—C5—H5	119.4	C14—C13—S1	119.90 (16)
C6—C5—H5	119.4	C15—C14—C13	119.7 (2)
C7—C6—C5	114.74 (17)	C15—C14—H14	120.1
C7—C6—H6	122.6	C13—C14—H14	120.1
C5—C6—H6	122.6	C14—C15—C16	119.0 (2)
C6—C7—O1	124.24 (17)	C14—C15—H15	120.5
C6—C7—C2	125.10 (17)	C16—C15—H15	120.5
O1—C7—C2	110.65 (16)	C17—C16—C15	121.8 (2)
C1—C8—O1	110.66 (17)	C17—C16—Cl1	118.80 (17)
C1—C8—C12	134.1 (2)	C15—C16—Cl1	119.43 (18)
O1—C8—C12	115.19 (18)	C16—C17—C18	119.1 (2)
C11—C9—C10	111.3 (2)	C16—C17—H17	120.5
C11—C9—C4	111.85 (18)	C18—C17—H17	120.5
C10—C9—C4	111.7 (2)	C17—C18—C13	119.8 (2)
C11—C9—H9	107.2	C17—C18—H18	120.1
C10—C9—H9	107.2	C13—C18—H18	120.1
C4—C9—H9	107.2		
			
O2—S1—C1—C8	−24.7 (2)	S1—C1—C8—O1	−179.01 (14)
O3—S1—C1—C8	−155.17 (18)	C2—C1—C8—C12	−177.8 (2)
C13—S1—C1—C8	90.33 (19)	S1—C1—C8—C12	3.0 (4)
O2—S1—C1—C2	156.20 (17)	C7—O1—C8—C1	−0.3 (2)
O3—S1—C1—C2	25.73 (19)	C7—O1—C8—C12	178.10 (17)
C13—S1—C1—C2	−88.77 (18)	C3—C4—C9—C11	−122.0 (2)
C8—C1—C2—C7	0.0 (2)	C5—C4—C9—C11	57.6 (3)
S1—C1—C2—C7	179.20 (14)	C3—C4—C9—C10	112.5 (2)
C8—C1—C2—C3	178.7 (2)	C5—C4—C9—C10	−67.9 (3)
S1—C1—C2—C3	−2.0 (3)	O2—S1—C13—C18	−146.81 (17)
C7—C2—C3—C4	−0.1 (3)	O3—S1—C13—C18	−15.87 (19)
C1—C2—C3—C4	−178.8 (2)	C1—S1—C13—C18	97.77 (18)
C2—C3—C4—C5	0.4 (3)	O2—S1—C13—C14	33.5 (2)
C2—C3—C4—C9	−179.98 (18)	O3—S1—C13—C14	164.47 (17)
C3—C4—C5—C6	−0.6 (3)	C1—S1—C13—C14	−81.89 (18)
C9—C4—C5—C6	179.76 (18)	C18—C13—C14—C15	−1.2 (3)
C4—C5—C6—C7	0.5 (3)	S1—C13—C14—C15	178.50 (17)
C5—C6—C7—O1	178.91 (16)	C13—C14—C15—C16	0.6 (3)
C5—C6—C7—C2	−0.2 (3)	C14—C15—C16—C17	0.2 (3)
C8—O1—C7—C6	−178.93 (18)	C14—C15—C16—Cl1	−179.07 (17)
C8—O1—C7—C2	0.3 (2)	C15—C16—C17—C18	−0.5 (3)
C3—C2—C7—C6	0.0 (3)	Cl1—C16—C17—C18	178.81 (17)
C1—C2—C7—C6	179.07 (18)	C16—C17—C18—C13	−0.1 (3)
C3—C2—C7—O1	−179.21 (16)	C14—C13—C18—C17	0.9 (3)
C1—C2—C7—O1	−0.2 (2)	S1—C13—C18—C17	−178.76 (16)
C2—C1—C8—O1	0.2 (2)		

### Hydrogen-bond geometry (Å, °)

**Table:**

Cg1 is the centroid of the C2–C7 benzene ring.

**Table:**

*D*—H···*A*	*D*—H	H···*A*	*D*···*A*	*D*—H···*A*
C12—H12C···O3^i^	0.96	2.57	3.489 (3)	160
C11—H11B···Cg^ii^	0.96	2.81	3.562 (3)	136

Symmetry codes: (i) *x*, −*y*+1/2, *z*+1/2; (ii) −*x*+1, *y*−1/2, −*z*+1/2.
